# The Fall and Rise of US Inequities in Premature Mortality: 1960–2002

**DOI:** 10.1371/journal.pmed.0050046

**Published:** 2008-02-26

**Authors:** Nancy Krieger, David H Rehkopf, Jarvis T Chen, Pamela D Waterman, Enrico Marcelli, Malinda Kennedy

**Affiliations:** 1 Department of Society, Human Development and Health, Harvard School of Public Health, Boston, Massachusetts, United States of America; Center for Tobacco Control Research and Education, United States of America

## Abstract

**Background:**

Debates exist as to whether, as overall population health improves, the absolute and relative magnitude of income- and race/ethnicity-related health disparities necessarily increase—or derease. We accordingly decided to test the hypothesis that health inequities widen—or shrink—in a context of declining mortality rates, by examining annual US mortality data over a 42 year period.

**Methods and Findings:**

Using US county mortality data from 1960–2002 and county median family income data from the 1960–2000 decennial censuses, we analyzed the rates of premature mortality (deaths among persons under age 65) and infant death (deaths among persons under age 1) by quintiles of county median family income weighted by county population size. Between 1960 and 2002, as US premature mortality and infant death rates declined in all county income quintiles, socioeconomic and racial/ethnic inequities in premature mortality and infant death (both relative and absolute) shrank between 1966 and 1980, especially for US populations of color; thereafter, the relative health inequities widened and the absolute differences barely changed in magnitude. Had all persons experienced the same yearly age-specific premature mortality rates as the white population living in the highest income quintile, between 1960 and 2002, 14% of the white premature deaths and 30% of the premature deaths among populations of color would not have occurred.

**Conclusions:**

The observed trends refute arguments that health inequities inevitably widen—or shrink—as population health improves. Instead, the magnitude of health inequalities can fall or rise; it is our job to understand why.

## Abstract 2

Nancy Krieger and colleagues found evidence of decreasing, and then increasing or stagnating, socioeconomic and racial inequities in US premature mortality and infant death from 1960 to 2002.

## Editors' Summary

**Background:**

One of the biggest aims of public health advocates and governments is to improve the health of the population. Improving health increases people's quality of life and helps the population be more economically productive. But within populations are often persistent differences (usually called “disparities” or “inequities”) in the health of different subgroups—between women and men, different income groups, and people of different races/ethnicities, for example. Researchers study these differences so that policy makers and the broader public can be informed about what to do to intervene. For example, if we know that the health of certain subgroups of the population—such as the poor—is staying the same or even worsening as the overall health of the population is improving, policy makers could design programs and devote resources to specifically target the poor.

To study health disparities, researchers use both relative and absolute measures. Relative inequities refer to ratios, while absolute inequities refer to differences. For example, if one group's average income level increases from $1,000 to $10,000 and another group's from $2,000 to $20,000, the relative inequality between the groups stays the same (i.e., the ratio of incomes between the two groups is still 2) but the absolute difference between the two groups has increased from $1,000 to $10,000.

Examining the US population, Nancy Krieger and colleagues looked at trends over time in both relative and absolute differences in mortality between people in different income groups and between whites and people of color.

**Why Was This Study Done?:**

There has been a lot of debate about whether disparities have been widening or narrowing as overall population health improves. Some research has found that both total health and health disparities are getting better with time. Other research has shown that overall health gains mask worsening disparities—such that the rich get healthier while the poor get sicker.

Having access to more data over a longer time frame meant that Krieger and colleagues could provide a more complete picture of this sometimes contradictory story. It also meant they could test their hypothesis about whether, as population health improves, health inequities necessarily widen or shrink within the time period between the 1960s through the 1990s during which certain events and policies likely would have had an impact on the mortality trends in that country.

**What Did the Researchers Do and Find?:**

In order to investigate health inequities, the authors chose to look at two common measures of population health: rates of premature mortality (dying before the age of 65 years) and rates of infant mortality (death before the age of 1).

To determine mortality rates, the authors used death statistics data from different counties, which are routinely collected by state and national governments. To be able to rank mortality rates for different income groups, they used data on the median family incomes of people living within those counties (meaning half the families had income above, and half had incomes below, the median value). They calculated mortality rates for the total population and for whites versus people of color. They used data from 1960 through 2002. They compared rates for 1966–1980 with two other time periods: 1960–1965 and 1981–2002. They also examined trends in the annual mortality rates and in the annual relative and absolute disparites in these rates by county income level.

Over the whole period 1960–2002, the authors found that premature mortality (death before the age of 65) and infant mortality (death before the age of 1) decreased for all income groups. But they also found that disparities between income groups and between whites and people of color were not the same over this time period. In fact, the economic disparities narrowed then widened. First, they shrank between 1966 and 1980, especially for Americans of color. After 1980, however, the relative health inequities widened and the absolute differences did not change. The authors conclude that if all people in the US population experienced the same health gains as the most advantaged did during these 42 years (i.e., as the whites in the highest income groups), 14% of the premature deaths among whites and 30% of the premature deaths among people of color would have been prevented.

**What Do These Findings Mean?:**

The findings provide an overview of the trends in inequities in premature and infant mortality over a long period of time. Different explanations for these trends can now be tested. The authors discuss several potential reasons for these trends, including generally rising incomes across America and changes related to specific diseases, such as the advent of HIV/AIDS, changes in smoking habits, and better management of cancer and cardiovascular disease. But they find that these do not explain the fall then rise of inequities. Instead, the authors suggest that explanations lie in the social programs of the 1960s and the subsequent roll-back of some of these programmes in the 1980s. The US “War on Poverty,” civil rights legislation, and the establishment of Medicare occurred in the mid 1960s, which were intended to reduce socioeconomic and racial/ethnic inequalities and improve access to health care. In the 1980s there was a general cutting back of welfare state provisions in America, which included cuts to public health and antipoverty programs, tax relief for the wealthy, and worsening inequity in the access to and quality of health care. Together, these wider events could explain the fall then rise trends in mortality disparities.

The authors say their findings are important to inform and help monitor the progress of various policies and programmes, including those such as the Healthy People 2010 initiative in America, which aims to increase the quality and years of healthy life and decrease health disparities by the end of this decade.

**Additional Information.:**

Please access these Web sites via the online version of this summary at http://dx.doi.org/10.1371/journal.pmed. 0050046.

- *Healthy People 2010* was created by the US Department of Health and Human Services along with scientists inside and outside of government and includes a comprehensive set of disease prevention and health promotion objectives for the US to achieve by 2010, with two overarching goals: to increase quality and years of healthy life and to eliminate health disparities
- Johan Mackenbach and colleagues provide an overview of mortality inequalities in six Western European countries—Finland, Sweden, Norway, Denmark, England/Wales, and Italy—and conclude that eliminating mortality inequalities requires that more cardiovascular deaths among lower socioeconomic groups be prevented, as well as more attention be paid to rising death rates of lung cancer, breast cancer, respiratory disease, gastrointestinal disease, and injuries among women and men in the lower income groups.
- The WHO Health for All program promotes health equity
- A primer on absolute versus relative differences is provided by the American College of Physicians

## Introduction

One new debate appearing in the public health literature is: as population health improves, do relative and absolute social inequalities in health widen or shrink [1–7]? An increasingly common view, typically drawing on recent data from the United States, is that relative, if not also absolute, health disparities are bound to increase as mortality rates decline, largely because groups with the most education and most resources are most able to take advantage of new knowledge and technology [1–3]. By contrast, others, reviewing contemporary data from other countries, such as Canada, posit that as mortality rates drop, health inequities tend to “flatten up” [4], largely because improvements in population health are driven by “‘pulling up' the health of the lower groups” (p. 592 [4]). Still others, examining both the North American and European data over longer spans of time, hold that no one general pattern can be expected; instead, the circumstances leading to improvements in population health and affecting the magnitude of health inequities are historically contingent and dependent on the societal context and its public health, political, and economic priorities [5–9].

Given the profound policy implications of this debate, rigorous examination of these hypotheses is warranted. If, for example, increased health inequities inevitably accompany improvements in population health, it would suggest that the focus on health inequities should be secondary to concerns about overall trends in the level of population health [2]. Conversely, if improvements in overall population health chiefly result from larger gains among those faring worst, it would suggest that as long as population health improves, health inequities should decline [4]. If, however, the relationship between population health and the magnitude of health inequities is more variable, it would imply that resources are needed to tackle both concerns. Reflecting this tension between needing to address both the level of overall population health and the magnitude of health inequities are the twin objectives of *Healthy People 2010*, which are to both “increase years and quality of healthy life” and “eliminate health disparities” [10]. Also warranting consideration are growing methodological discussions about the measurement of health inequities and whether relative or absolute inequities—and relative or absolute declines—should be the focus of concern [11,12]. The question as to choice of relative or absolute measures arises because as rates for any given health outcome decline, it is conceivable that faster-falling rates in one group compared to another could lead to an increase in the relative risk for that outcome, albeit reflecting a smaller absolute difference compared to when rates in both groups were higher. Whereas from an etiologic perspective a focus on relative risks might be most appropriate, from a public health standpoint a reduction in absolute risk is vital [11,12].

To date, however, US research on the changing magnitude of health inequities has provided only a partial picture, largely because of limited data and truncated timeframes. Studies typically have focused on racial/ethnic, and especially black/white, health inequities (e.g., [13–18], largely due to unavailable or inconsistent socioeconomic data in US birth and death certificates, until 1968 and 1989, respectively [19,20]. These studies of racial/ethnic mortality inequities, moreover, have chiefly examined trends since the 1980s, mainly in relation to relative differences, with a few also comparing rates at the beginning versus end of a longer time period (e.g., 1960 versus 2000) [13–18]. The smaller body of research examining trends in US socioeconomic inequities in mortality has likewise focused on more recent periods, e.g., since 1980, or else has gone back to at most 1968 (e.g., [21–29]), which is when the conventional US public-release mortality data begin [30]. Some research has also compared post-1969 data [29] to results from Kitagawa and Hauser's classic 1960 study on socioeconomic differentials in US mortality [31], albeit lacking data for the intervening years (i.e., 1961–1968). Together, these two strands of work on racial/ethnic and socioeconomic inequities in US mortality have tended to support the hypothesis that as overall death rates have declined, social inequities in mortality have increased.

Suggesting, however, that a more complex picture might exist are a handful of studies documenting that, during the mid-to late-20th century, relative, and in some cases absolute, US black/white disparities in infant mortality [32], avoidable mortality [6], age-specific mortality [33], and life expectancy [34] have variously shrunk and widened. A recent study conducted in New York City, moreover, found that in a context of declining death rates, comparing the period 1989–1991 versus 1999–2001, relative socioeconomic inequalities in mortality persisted but declined for all-cause mortality and in years of person-life lost before age 65, stayed the same for several leading causes of death (e.g., cardiovascular disease, cancer), and increased for HIV/AIDS [35].

We accordingly decided to test the hypothesis that health inequities necessarily widen—or shrink—in a context of declining mortality rates, by examining annual US mortality data spanning from 1960 to 2002. We opted to use methods that would allow us to analyze both relative and absolute changes in health inequities as well as changes in slopes over time. We chose the time period starting in 1960, because unlike analyses focused on trends since 1969, it precedes as well as encompasses the period of the mid-1960s, a time when new federal policies were enacted with the intent of reducing socioeconomic and racial/ethnic inequalities, overall and also in relation to medical care [6,9,36–42]. Examples include the various federal policies constituting the “War on Poverty,” the 1964 US Civil Rights Act, and the establishment of Medicare, Medicaid, and community health centers [32,33,36–42]. The selected timeframe likewise encompasses subsequent periods of active debate and change regarding government policies and spending on antipoverty and civil rights initiatives [32,33,36–42]. Moreover, recognizing the complex interplay between socioeconomic and racial/ethnic inequality and health inequities in the United States [8,16,17], we opted to analyze trends in socioeconomic inequities in premature mortality for the total population and by race/ethnicity. By conducting these analyses, we reasoned we would be able to see often overlooked patterns of socioeconomic inequities within racial/ethnic groups as well as better identify which groups experienced the greatest changes in both premature mortality rates and inequities in these rates. Our expectation was that by expanding the temporal frame, methods of analysis, and populations studied, we would be able to generate a more complete picture of temporal changes in US inequities in mortality. Our a priori prediction was that the societal changes of the mid-1960s would be embodied [8] and manifested in reductions in socioeconomic and racial/ethnic health inequities that preceded the documented [13–17,21–29] post-1980 widening of health disparities.

The primary outcome we chose for our analyses was premature mortality, defined as the age-standardized death rate for persons under age 65. We chose this measure because, unlike life expectancy [43,44] and years of person-lives lost [45,46], it is easy to understand, easy to compare, methodologically transparent, and a sensitive indicator of inequities in health status and health care [47,48]. Both longitudinal and cross-sectional research in the United States and the United Kingdom have shown that premature mortality is socially patterned, with early death most common among populations confronted by economic deprivation and racial discrimination [5,7,21,28]. We additionally focused on the infant death rate, as a subcomponent of premature mortality, both because infant mortality is a well-accepted measure of population health and because it might exhibit greater temporal responsiveness to concurrent societal changes [5,9,22,32]. We accordingly sought out data to analyze rates of premature mortality and infant death among US counties, ranked by income level, for the period 1960–2002, both for the total population and by race/ethnicity. We chose age 65 as the cut-off point for premature mortality since this age determines eligibility for Social Security and Medicare [40], and because using the current US definition of premature mortality as death before age 75 [10] would be inappropriate because an average life expectancy of at least 65 years was consistently attained by US black men only in 1995—compared to 1944 for the total US and white population, 1938 and 1946 for white women and white men, 1954 for black women, and 1973 for the black population overall [49].

## Methods

### Population: Deaths and Denominator

We obtained US county 1960–1967 mortality data from the US National Center for Health Statistics (NCHS) [50], for which we then manually located and identified the correct county code for each of the 3,073 counties; we extracted the available 1968–2002 data from the NCHS US Compressed Mortality Files [30]. Denominators for 1970–2000 consisted of the US Census decennial counts and intercensal population estimates and the NCHS interpolated estimates for 1968 and 1969 and extrapolated estimates for 2001 and 2002 [30]; using the 1960 and 1968 population data, we estimated the 1961–1967 denominators using linear interpolation. All analyses and data use were approved by the Harvard School of Public Health Human Subjects Committee (Protocol #P12481–01).

The only racial/ethnic categories available in the US mortality data for the full time period under study were “white,” “Negro” (or “black”), and “other” [30,50]. We accordingly distinguished solely between the US white population and US populations of color, given the persistence of what W. E. B. Du Bois in 1904 famously termed the US “colorline” [51], which divides the racially dominant US white population and US populations of color (as also reflected in the inconsistent racial/ethnic categories employed for the latter group in the 1960–2000 US decennial census [16–19,30,52,53]). New Jersey death certificates did not identify race/ethnicity in 1962 and 1963, precluding use of these two years' data (comprising only 3% of the US population). Alaska analyses before 1989 were for the entire state only (equaling 0.01%–0.02% of the US population) because the pre-1989 population and mortality data employed nonidentical county boundaries.

### County Economic Resources

Because of the loss of the computerized 1960 census “100% detail” file and its household economic data (Marie Pees, US Census Bureau, personal communication, 8 June 2005), we could only analyze available pretabulated 1960 county socioeconomic data. We located the 1960 census county median family income data [54] and obtained analogous data from the 1970–2000 census [52,53]. These data were highly correlated (*r* ≥ 0.97 for 1980–2000) with the more commonly used county median household income data (which includes all types of households, not just those composed of family members related by blood or marriage). To normalize the income and cost-of-living data across regions, we re-expressed the county median family income data using the US 2000 census referent of 1999 dollars, adjusted by the Consumer Price Index (Urban, All Items)(CPI-U) [55]. We interpolated the county median family income data for intercensal years and extrapolated for 2001 and 2002 based on the slope for 1990–2000. We then assigned counties to quintiles of median family income weighted by county population size, given enormous variation in county population size. We provide the cut-off points for and maps of the county median family income quintile distribution for decennial years 1960–2000 in Figures S1–S5 and Table S1.

### Statistical Analysis

For each calendar year, we aggregated the county mortality and population data in each county income quintile and calculated each quintile's age-standardized premature mortality rate (deaths before age 65, standardized to the US 2000 standard million) and infant death rate (deaths among persons under age 1). We used SAS 8.1 to conduct all of our analyses [56], except where indicated otherwise. For 1960–1987, given data limitations, we used the NCHS algorithm to calculate the infant death denominator (i.e., multiplied “the population in the 1–4 age category by 0.25”; p. 9, [30]). For each outcome, we then compared rates in the lower to highest county income quintiles to calculate each year's age-standardized incidence rate ratio (IRR), as a measure of the relative disparity (the metric used in most research on this topic), and also the incidence rate difference (IRD), a measure of absolute difference, since the absolute gap is more relevant for assessing the actual population burden of mortality [5,7,11,12,31].

Reflecting the importance of considering different metrics of health inequities [11,12], we additionally tested our a priori hypothesis about changing rates of decline by investigating if the slope of the decline for premature mortality and infant deaths, especially in the lower income quintiles, was steepest in the 1966–1980 time period, compared to both 1960–1965 and 1981–2002. We then conducted post-hoc confirmatory analyses using joinpoint regression techniques to identify empirically detectable inflection points in the average annual percent change in these rates [57], noting that prior research on trends in US inequities in overall mortality has not, to our knowledge, tested for changes in slopes or inflection points. In these models, also called segmented line regression models, line segments are joined at points called “joinpoints.” When fit on the log scale, the slope of the line segments are interpretable as the annual percent change in the rate, while the joinpoints denote statistically significant changes in the time trend [57]. As a further test of our study hypotheses, we additionally employed joinpoint analyses to test for changes in slopes or inflection points, over time, for the IRR and IRD for each of the four lower county income quintiles, comparing each quintile separately to the highest county income quintile. We conducted these analyses for the total population and stratified by race/ethnicity.

We also calculated each year's population attributable count and population attributable fraction (PAF) [58], respectively defined as the total and proportion of premature and infant deaths that would not have occurred if residents of the four lowest county income quintiles experienced the same yearly age-specific death rates of persons residing in the highest county income quintile; a related set of calculations set as referent group the mortality rates of white persons in the highest county income quintile. The PAF can be meaningfully interpreted as an excess fraction expressing the gap between the empirically observed and the then achievable deaths rates across quintiles of county median family income [31,58]. We caution that in our analyses, the PAF cannot be interpreted in directly causal terms: it would be incorrect to infer that if persons in the lowest county income quintile suddenly had the same higher incomes as persons in the highest county income quintile, they would immediately have the same lower mortality rate. Such a scenario would be implausible on several grounds, including: (a) lifecourse (a change in adult circumstances may not necessarily reverse harm done in earlier life); (b) etiologic period (especially for chronic diseases); and (c) recognition that altering societal determinants of health requires addressing not only income but other societal, medical, and public health determinants as well [2,5,7,8]. Nevertheless, as employed in our analyses, the PAF provides an appropriate and useful measure of preventable excess mortality.

Future analyses will examine, in more detail, the socioeconomic trends in premature mortality by geographic region and also by gender, age group, leading cause of death, birth cohort, and, for the year 2000 data, by more refined racial/ethnic categories. By contrast, the purpose of the present analyses was specifically to test the hypothesis that as premature mortality rates decline, socioeconomic inequities in premature mortality either widen or shrink.

## Results

**Figure 1:**
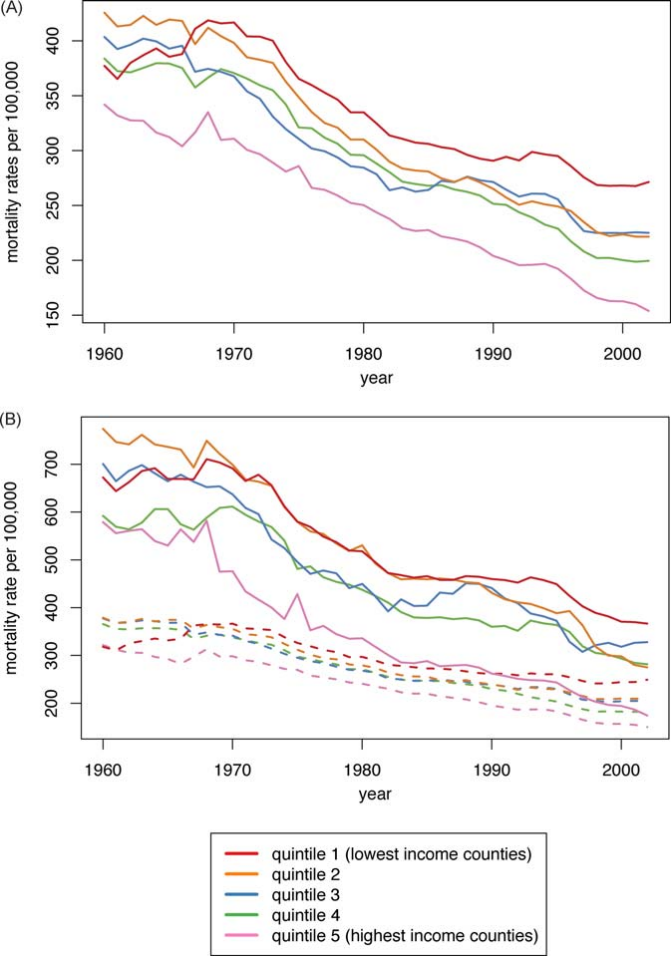
Premature Mortality Rates by Income and Race/Ethnicity Premature mortality rates (deaths under age 65), United States, 1960–2002, by county median family income quintile (A); and for the US white population (dashed lines) and populations of color (solid lines) (B).

**Figure 2:**
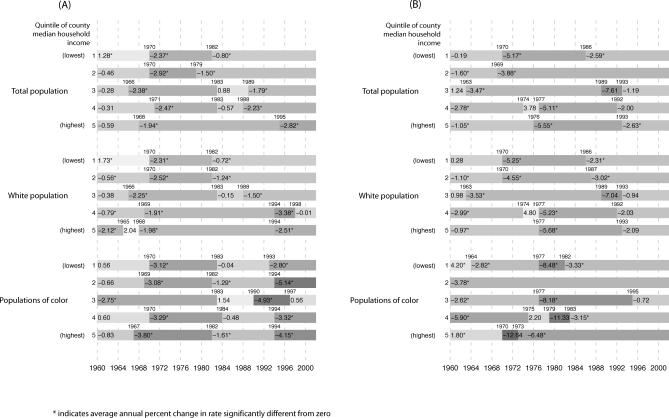
Changes in Premature Mortality and Infant Death Rates by Income and Race/Ethnicity Estimated average annual percent change in: the age-standardized premature mortality rate (deaths under age 65) (A), and the infant death rate (B), and location of statistically significant joinpoints by quintile of county median household income for total population, white population, and populations of color, United States, 1960–2002.

**Figure 3:**
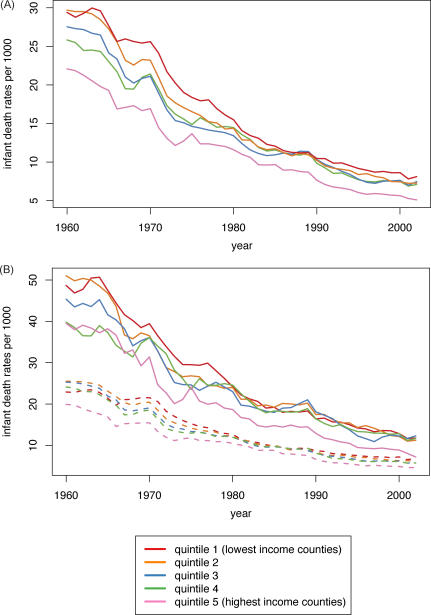
Infant Death Rates by Income and Race/Ethnicity Infant death rates by county median family income quintile, United States, 1960–2002 (A); and for the US white population (dashed lines) and populations of color (solid lines) (B).

In accord with our a priori hypothesis, between 1960 and 2002, as US premature mortality and infant death rates declined in all county income quintiles, and absolute and relative socioeconomic inequities in premature mortality shrank between 1966 and 1980, especially for US populations of color; thereafter, the relative health inequities widened and the absolute inequities stagnated (Figures 1–3; Tables 1–8). In 1970 and 1980, the relative difference in premature mortality, comparing the populations in the lowest to highest county income quintiles was 1.3 (95% confidence interval [CI] = 1.3,1.4), and the absolute difference declined (in death rates per 100,000) from 106 (95% CI = 103,109) to 85 (95% CI = 82,87); in 2000, the relative difference equaled 1.6 (95% CI = 1.6,1.7) and the absolute difference had climbed back to 105 (95% CI = 103,107) (Tables 1 and 2). Between 1966 and 1980, the average annual change in the premature mortality rate in the lowest income quintile was 6.2 deaths per 100,000, but in 1981–2002 it declined to only 3.0 per 100,000; by contrast, in the highest income quintile this rate of decline changed from only 5.1 to 4.3 per 100,000 (Tables 1 and 2).

**Table 1:**
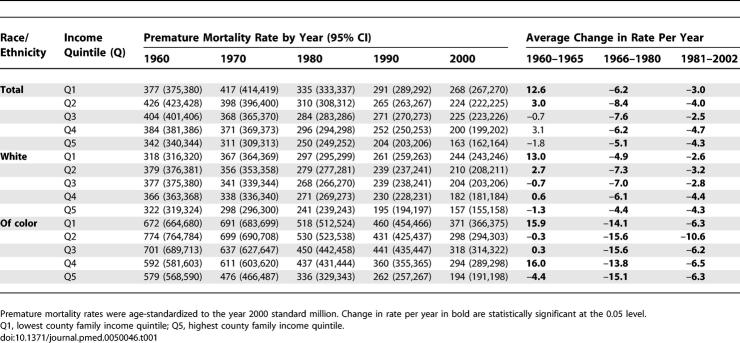
US Premature Mortality Rates (Deaths before Age 65) per 100,000

**Table 2:**
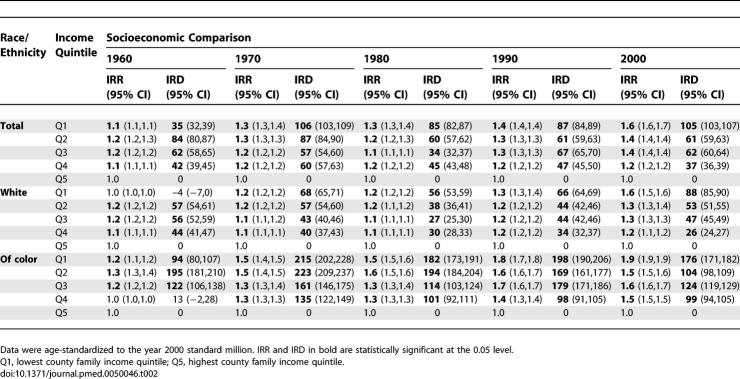
US Premature Mortality Incidence Rate Ratio (IRR) and Incidence Rate Difference (IRD, Cases per 100,000)

Post-hoc analyses confirmed that statistically significant (*p* < 0.05) inflection points occurred in the average annual percent change in these rates, especially in the lower income quintiles, during the latter part of the 1960s (largest decline) and the early part of the 1980s (smallest decline) (Figure 2A). For example, in the lowest income quintile, the inflection points identified were for 1972 and 1980; during this time period, the average annual percent change in premature mortality rates was 2.37%, but after 1980 it was only 0.80% (Figure 2A). By contrast, among the population in the highest income quintile, the inflection points identified were for 1968 and 1995; between these two points, this group experienced a 1.94% annual percent change in premature mortality rates, which then increased to a 2.82% change (Figure 2A). Amounting to a quarter-century lag, it was not until 2002 that premature mortality rates in the lowest county income quintile equaled those attained in 1975 by the highest county income quintile (Figure 1A). Reflecting these quintile-specific changes in rates, joinpoint analyses of the IRR and IRD over time comparing each of the four lower county income quintiles to the highest county income quintile provided further evidence—as shown by the presence of statistically significant inflection points (*p* < 0.05)—that between the mid-1960s and early 1980s inequities in premature mortality lessened, after which they increased, with these patterns typically strongest in the lower county income quintiles (Table 7).

This overall picture obscures concurrent—yet changing—racial/ethnic disparities within county income quintiles and economic disparities within racial/ethnic groups (Figure 1B; Tables 1 and 2). Between 1960 and 2002, premature mortality rates within each county income quintile among populations of color exceeded those of their white counterparts, with the excess diminishing over time. For example, in 1970, the premature mortality rates in the lowest income quintile (per 100,000) was 691 (95% CI = 683,699) for the populations of color but 367 (95% CI = 364,369) for the white population, a 1.8-fold relative difference and an absolute difference of 324 per 100,000 (i.e., nearly the premature mortality rate for the white population itself) (Tables 1 and 2). Within the highest income quintile, the premature mortality rates were 476 (95% CI = 466,487) for the populations of color and 298 (95% CI 296,300) for the white population, a 1.6-fold relative difference and a smaller absolute difference of 178 per 100,000 (Tables 1 and 2). In 2000, among persons in the lowest income quintile, the relative and absolute difference of premature mortality rates among populations of color (371 per 100,000; 95% CI = 366,375) compared to the white population (244 per 100,000; 95% CI = 243,246) equaled 1.5 and 127 per 100,000, respectively (Tables 1 and 2). Among those in the highest income quintile, the respective comparisons of the premature mortality rate among populations of color (194 per 100,000; 95% CI = 191,198) and the white population (157 per 100,000; 95% CI = 155,158), which translated to a relative difference of 1.2 and absolute difference of 37 per 100,000 (Tables 1 and 2). Notably, parity in rates of premature mortality were attained by US populations of color only for those in the highest county income quintile: they first equaled those of whites in the lowest county income quintile in the mid-1980s and only equaled those of whites in the second highest county income quintile in 2001 (Figure 1B).

As shown by analysis of the absolute rates (Table 1) and the average annual percent decline in these rates (Figure 2A), the socioeconomic gaps in premature mortality among both the white population and the populations of color narrowed between the mid-1960s and the early 1980s. During this time period, the greatest declines in US premature mortality rates occurred among US populations of color, especially in the lowest two county income quintiles. For example, in analyses using the a priori temporal cut-off points of 1966 and 1980, between these two time points, the average annual change in the premature mortality rate among the populations of color was between 13 and 15 per 100,000 in all income quintiles, but declined to between 6 and 11 per 100,000 between 1981 and 2002; the corresponding declines for the white population were between 4 and 7 per 100,000, and then 2 and 5 per 100,000 (Table 1). Figure 2A shows the results of the joinpoint regression analyses, which detected significant inflection points occurring both between 1965 and 1970 and again between 1982 and 1984. These joinpoint analyses indicated that after the early 1980s, the average annual percent change in premature mortality rates dropped to less than half that of the preceding period for all socioeconomic-racial/ethnic strata – except for the white population living in the two highest county income quintiles, whose rate of decline stayed the same or increased.

Consequently, had the same annual rate of decline in premature mortality deaths rates observed in each county income quintile between 1966 and 1980 extended until 2002, then in 2002, the premature death rate in the lowest county income quintile would have been 16.4% lower (observed versus predicted rate of 271.4 versus 226.8 per 100,000), while that in the highest county income quintile would have been 13.6% higher (observed versus predicted rate of 150.0 versus 174.9 per 100,000), respectively reflecting the post-1980 slowdown versus acceleration in the decline of the premature mortality rate in the lowest versus highest income quintile. The analogous comparisons by race/ethnicity for the lowest income quintile were, for the white population, a 15.8% excess (2002 observed versus predicted: 249.1 versus 209.7 per 100,000) and for the populations of color, a 15.2% excess (2002 observed versus predicted: 366.7 versus 310.9 per 100,000); for the highest income quintile, they were, for the white population, a deficit of 16.4% (2002 observed versus predicted: 150.2 versus 174.8 per 100,000), and for the populations of color, a smaller 8.1% excess (2002 observed versus predicted: 174.2 versus 160.1 per 100,000).

The infant death data provide a complementary picture of shrinking socioeconomic inequities (relative and absolute) before 1980, followed by their widening or stagnating thereafter (Figures 2B, 3A, and 3B; Tables 4, 5, and 8). Between 1960 and 2002, infant death rates substantially declined in every county income quintile, overall and by race/ethnicity, with the sharpest reductions in the absolute rates occurring between 1966 and 1980, especially among the populations of color in the lower income quintiles (Tables 4 and 5). The joinpoint analyses of the infant death rates indicated the sharpest declines, with significant inflection points, commenced in 1969–1970 for the populations in the lowest income quintiles and in the mid-1970s for those in the higher income quintiles (Figure 2B), with these trends more apparent in the white compared to the populations of color. In virtually all socioeconomic–racial/ethnic strata, however, additional inflection points detected in the mid-1980s indicated a significant slowing of the decline in the infant death rate, with the slowdown more pronounced among the populations of color compared to the white population (Figure 2B). For example, in the lowest income quintile, the average annual percent decline among the white population changed from 5.3% for 1970–1986 to 2.3% for 1986–2002; among populations of color, it changed from 8.5% for 1977–1982, to 3.3% for 1982–2002 (Figure 2B). Analogous patterns were evident for the joinpoint analyses of the IRR and IRD for the infant death rates over time (Table 8). Specifically, statistically significant (*p* < 0.05) inflection points marking a lessening of inequities occurred in the mid-1960s to early 1970s; those indicating a widening of inequities occurred thereafter (Table 8).

**Table 3:**
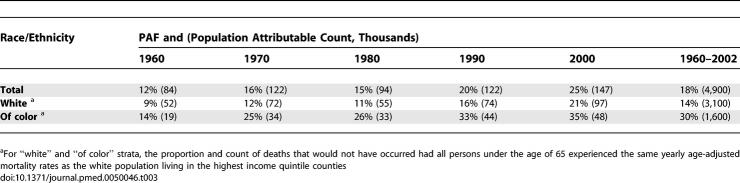
US Premature Mortality Population Attributable Fraction (PAF) and Population Attributable Count (in Thousands) for Deaths in Lower Four Compared to Highest County Income Quintile

**Table 4:**
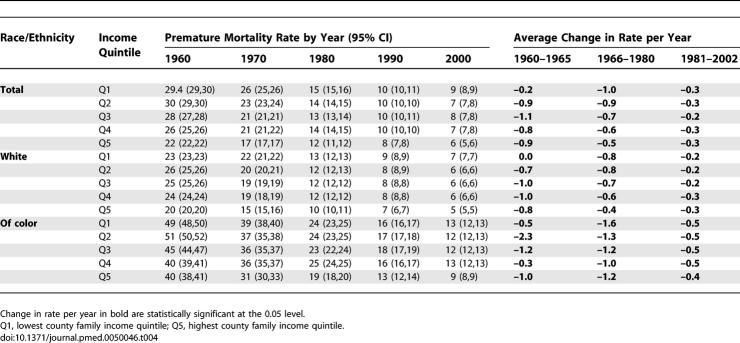
Infant Death Rates (per 1,000 Persons under Age 1)

**Table 5:**
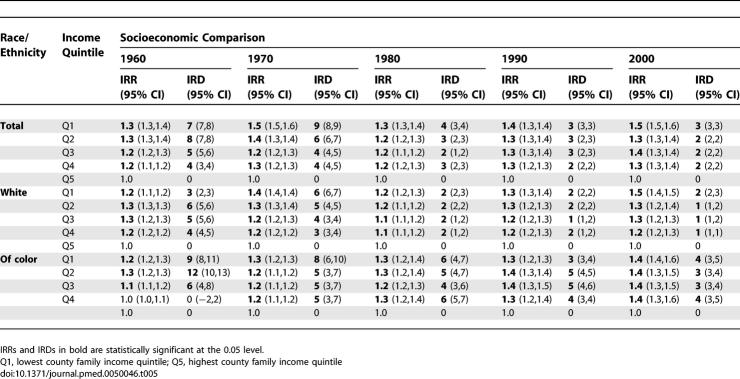
Infant Death Incidence Rate Ratio (IRR) and Incidence Rate Difference (IRD, per 1,000 Persons under Age 1)

Only in 2002 did the infant death rate among populations of color in the highest county income quintile attain parity with that of whites in the lowest county income quintile. Had the same annual rate of decline in infant deaths rates observed in each county income quintile between 1965 and 1980 extended until 2002, then in 2002, the infant death rate in the lowest county income quintile would have been 39.2% lower (observed versus predicted rate of 8.1 versus 4.9 per 1,000), while that in the highest county income quintile would have been 4.7% higher (observed versus predicted rate of 5.1 versus 5.3 per 1,000), again reflecting the post-1980 slowdown versus acceleration in the decline of the infant death rate in the lowest versus highest income quintile. The analogous comparisons by race/ethnicity for the lowest income quintile were, for the white population, a 41.2% excess (2002 observed versus predicted: 6.9 versus 4.1 per 1,000) and for the populations of color, a 32.6% excess (2002 observed versus predicted: 11.8 versus 7.9 per 1,000); for the highest income quintile, they were, for the white population, a deficit of 4.9% (2002 observed versus predicted: 4.6 versus 4.8 per 1,000), and for the populations of color, a smaller 6.6% excess (2002 observed versus predicted: 7.2 versus 6.7 per 1,000).

The differential reductions in premature mortality and infant deaths rates by county income quintiles during the study period translated to an excess fraction of 4.9 million lives cut short (Table 3). Included among these were the deaths of an estimated 460,000 infants (Table 6), equaling 20% of all infant deaths. Similarly, 18% of the premature deaths that occurred between 1960 and 2002 would have been averted had the populations in the bottom four quintiles experienced the same yearly age-specific premature mortality rates as the population in the highest income quintile (Table 3). Moreover, had all persons experienced the same yearly age-specific premature mortality rates as the white population living in the highest county income quintile, then between 1960 and 2002, the proportion of premature deaths that would not have occurred equaled 14% for the white population but 30% for the populations of color (Table 3).

**Table 6:**
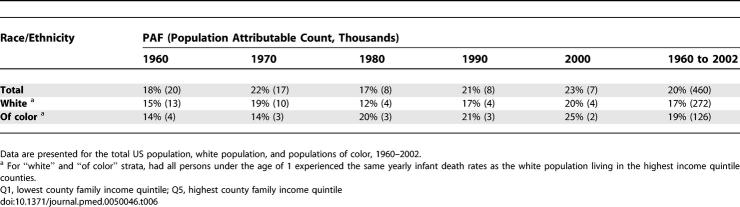
Infant Death Population-Attributable Fraction (PAF) and Population Attributable Count (in Thousands) for Deaths in Lower Four Compared to Highest County Income Quintile

**Table 7:**
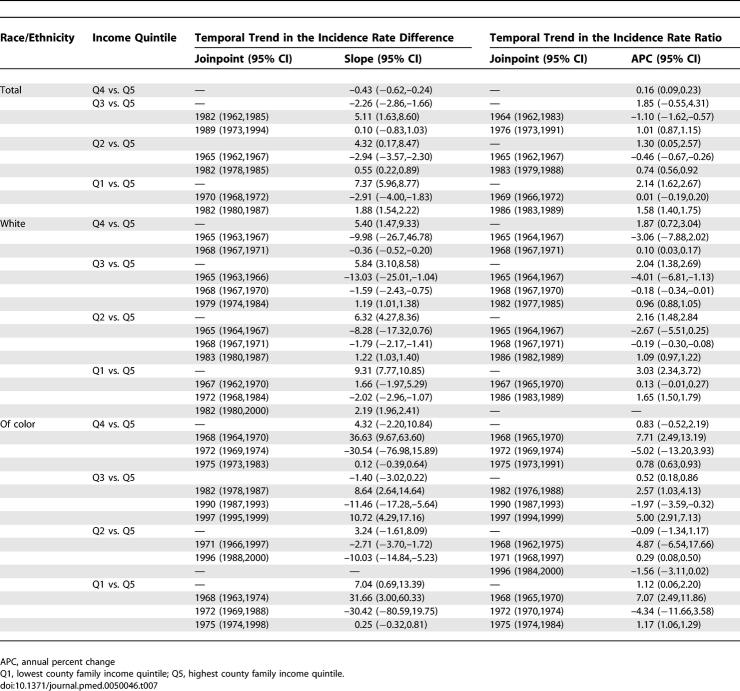
Joinpoint Models for the Temporal Trend in Premature Mortality Rate Difference (per 100,000 under age 65) and Incidence Rate Ratio

**Table 8:**
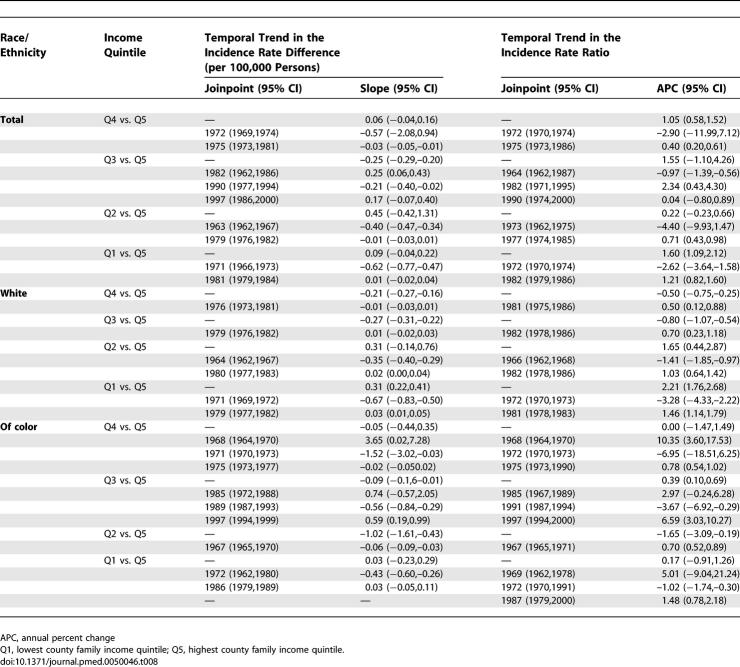
Joinpoint Models for the Temporal Trend in Infant Death Rate Difference (Deaths per 1,000 Persons under age 1) and Death Rate Ratio

## Discussion

Our results refute the hypotheses that as population health improves, health inequities necessarily either increase [1–3] or decline [4]. By extending the timeframe of analysis and by examining changes in slopes and inflection points, our analysis of US premature mortality and infant death data for 1960 to 2002 provides evidence of two distinct patterns: (1) between 1966 and 1980, the relative and absolute socioeconomic disparities in premature mortality and infant deaths shrank, overall and especially among US populations of color; and (2) thereafter, starting in 1981, and as previously documented [21–29], the relative and absolute socioeconomic gaps for premature mortality widened, while the relative gap for infant deaths remained unchanged and the absolute difference only marginally lessened. The net implication is that the societal patterning of socioeconomic inequities in mortality within and across racial/ethnic groups is historically contingent: context matters [5–9,59].

The observation that risk of premature mortality increases with economic deprivation and racial inequality obviously is not new; research documenting these social facts easily extends back to the late 18th century [5,59]. What our results newly underscore is that contemporary US inequities are not immutable. Comparing the results for 1966–1980 versus 1981–2002, the trends in the former timeframe give grounds for hope; the latter augur poorly for the *Healthy People 2010* objective of eliminating US socioeconomic and racial/ethnic health disparities [10].

### Study Limitations

Before offering interpretations of our key finding of declining—then increasing—inequities in premature mortality and infant death, several caveats require consideration. Relevant to causal inference [60], our study has several limitations. These include: (1) our necessary reliance on a repeat cross-sectional analytic design employing county-level mortality and socioeconomic data; (2) potential problems affecting the accuracy of the numerator and denominator data; and (3) the need for caution in linking the timing of observed inflection points to contemporaneous events, due to issues involving lag time and cohort effects.

One possibility is that our results might be an artifact of inaccurate numerator or denominator data. Despite federal reports indicating that 99% of all US deaths and births since 1960 have been registered [19], some local studies suggest that under-reporting of infant births and infant deaths occurred in rural and impoverished counties during the 1960s through mid-1970s, especially among African Americans [61,62]. Such selective underreporting could help explain why in the very early 1960s, among both the white population and populations of color, we found that rates of infant death and premature mortality were initially lower in the lowest compared to next two lowest quintiles. However, had the true premature mortality rates in the early-to-mid 1960s been higher than what we observed among populations in the lower income quintiles, and especially populations of color, then the magnitude of the absolute and relative decline in premature mortality rates from the beginning of the study period to 1980 would have been even greater—suggesting that our results, if anything, are conservative. Additionally, underreporting of infant births and deaths was effectively eliminated by 1980 [19,62] and so would not explain the observed post-1980 widening of socioeconomic inequities.

Also of concern is the US census undercount, which disproportionately has affected lower income populations and populations of color [63]. This undercount, however, declined considerably between 1960 and 2000, from 3.1% to 0.1% for the total population, and from 6.6% to 2.8% for the black population [63]. The net result would be to reduce, not inflate, the more recent estimates of social disparities in mortality, and hence not account for the observed post-1980 increase in health inequities. Results are also unlikely to be affected by racial/ethnic misclassification, given the broad groupings employed. Rather, demographic trends should have lowered risk of premature mortality among US populations of color, given the secular proportional decline in the US African American population (from 92% of US populations of color in 1960 to 72% in 2000 [52]) and the increase in foreign-born US populations of color, especially Latinos, and their associated “healthy immigrant” effect [16,17,64]. We further note that: (a) because of previously mentioned data limitations affecting racial/ethnic classification in the mortality data, we cannot restrict the “white” population to only “white non-Hispanic,” and (b) classification of Hispanics in the US census data, i.e., the denominators, began only in 1970 [65,66]. Research indicates, however, that a high proportion of US persons of Latin American ancestry choose the “race” category of “other” [65,66], so would be included within our category of “populations of color.” To the extent that this occurred, it would reduce risk of premature mortality within this group. Conversely, to the extent that Latinos were included in the “white” category, they would most likely be included in the lower county income quintiles [65,66], so would not account for the findings of the most quickly improving premature mortality rates in the upper county income quintiles.

Additional limitations on causal inference are imposed by our reliance on county-level mortality and socioeconomic data and our repeat cross-sectional analytic design. First, it would be erroneous to consider county-level income data as a “proxy” for individual- or family-level income data or to assume that use of county-level income data would lead to a conservative estimate of health inequities (e.g., due to the smaller range in median county level income levels compared to individual-level income levels). Instead, ecologic fallacy could arise if we attempted to infer individual-level associations from group-level correlations and, at the county level, this type of fallacy could potentially affect estimates of temporal trends [67]. We note, however, that the direction and magnitude of our observed socioeconomic and racial/ethnic trends in premature mortality and infant death are consistent with previous individual-level follow-up studies documenting increasing socioeconomic disparities in premature mortality in the 1980s [28] and decreasing black/white gaps in infant mortality in the mid-1960s [32]. These similar findings imply that our results are not unduly compromised by ecologic fallacy.

Second, caution also must be used in linking the timing of observed inflection points to contemporaneous events, because even though some major causes of premature mortality and infant death are influenced by concurrent socioeconomic position (e.g., fatal injury, homicide, and preventable deaths due to inadequate medical care), others may reflect the impact of lifetime socioeconomic deprivation (including, for infant deaths, on the fetus' mother, preconception) [5–9]. Also potentially of concern are birth cohort effects: whereas persons dying before the age of 65 in 1970 were, by definition, born between 1905 and 1969, those dying before the age of 65 in 2000 were born between 1945 and 1999 and thus belonging to divergent historical generations, with different health profiles. The observation of similar trends for the premature mortality and infant death analyses, however, provides some indication that contemporaneous conditions were contributing to shaping the social patterning of mortality; otherwise, if these rates and the observed disparities were driven chiefly by birth cohort and/or early life experiences (of either the decedents or their mothers, preconception), we arguably would have seen a greater disjuncture in the patterns observed for the premature mortality and infant death rates.

### Interpretation

The central objective of our study was to address current debates over whether, as population health improves, health disparities necessarily widen or shrink [1–7], and to do so by assessing the social patterning of absolute and relative socioeconomic inequities in US premature mortality and infant death rates over the past half-century. Assuming our results are plausible, the parallel findings for both premature mortality and infant death—that inequities shrank then widened—supports the view that the societal patterning of health inequities is historically contingent [5–9]. By better establishing the patterns that need to be explained, these findings stand as a preliminary but necessary first step for future testing of hypotheses that could account for the observed trends.

While any specific explanation of the observed trends is necessarily speculative, given both the intent of our study (i.e., testing whether, as population health improves, health inequities widen or shrink), and the limits on causal inference permitted by our study design, some tentative interpretation is warranted. At issue are both temporal changes in general societal factors, e.g., changing poverty levels, potentially affecting many causes of death [5–8,16,17,21–29] and disease-specific trends [5,34,35].

One plausible account would accordingly suggest the findings reflect the embodiment [8] of not only overall economic trends but also the health impacts of policies, programs, and priorities of both public and private institutions, in and outside of the health sector [5–9,31–42,59]. One candidate explanation for the observed overall decline in premature mortality rates in all income quintiles is the rising US per capita gross domestic product (GDP), which grew by 32% for 1961–1970, 23% for 1970–1980, 25% for 1980–1990, and 22% for 1990–2000 [68]. The continued rise in the per capita GDP, however, cannot explain the observed pattern of a diminishment and then increase or stagnation in the socioeconomic gradient. Nor can the observed trends of a shrinking then widening gap in premature mortality rates simply reflect fluctuations in the business cycle, given that periods of economic growth and recession occurred within both the 1966–1980 and 1981–2002 time periods [68,69]. It is similarly unlikely that chiefly individual-level behavioral factors can explain the faster then slower declines in premature mortality rates among persons in the lower county income quintiles, unless the argument can be made that health promotion efforts were more successful among persons in lower-income counties in the earlier compared to later time periods, which is doubtful.

It is also possible that disease-specific trends could account for some temporal segments of the observed changes in premature mortality inequities. In the case of HIV/AIDS, for example, its increasing concentration as a disease among US impoverished populations and populations of color [70,71], coupled with the introduction of antiretroviral treatments in the mid-1990s and inequities in access to these effective treatments [71], could in part contribute to the recent more quickly declining rates of premature mortality in more affluent compared to poorer counties. The changing societal patterning of HIV/ADS inequities in incidence, treatment, survival, and mortality, however, would not account for the observed pre-1980 reduction of premature mortality inequities, which predates HIV/AIDS' emergence in 1981 [70,71], nor is it likely to explain the more recent trends in inequities in infant deaths (since HIV/AIDS in the US remains predominantly a disease among adults). A recent study, moreover, found evidence of pronounced social inequities in US county mortality rates among persons 15–60 years old even when HIV/AIDS and homicide were excluded from the causes of death [18]. Similarly, while the increasing concentration of smoking, since the 1980s, among US working-class populations might contribute to more recent inequities in premature mortality (taking into account relevant—and different—lag times for smoking-related cardiovascular disease and cancer) [72,73], population patterns of smoking are unlikely to explain the shrinking premature mortality inequities in the pre-1980 period. The epochal US Surgeon General's report on smoking, for example, was published only in 1964, and it took over a decade before its publication had a notable impact on smoking rates [73,74]. Explanations concerning improved detection and treatment for cancer and cardiovascular disease, coupled with social inequities in access to these medical advances [75,76], would likewise have greater plausibility for the more recent versus earlier trends in premature mortality inequities that we observed. Trends in mortality due to these diseases, which predominantly affect adults, however, would not account for the trends in inequities we observed for infant deaths.

Additional, albeit conjectural, explanations might instead involve two key sets of societal determinants of health and health care: economic priorities and civil rights. Likely contributing to the 1966–1980 improvements are the positive impact of the “War on Poverty” and the civil rights legislation that expanded economic opportunity and resources and availability of health services, for both impoverished populations and populations of color, especially African Americans [32,33,40–42,77,78]. The parallel trends in reductions of premature mortality and infant death rates suggest an impact across all age groups, one suggestive of a period effect involving both changing societal conditions and new increased access to medical care for then very under-served populations. Conversely, the subsequent slowdown in reduction of health inequities, marked by the significant changes in inflection points in the early 1980s, is unlikely to reflect solely more affluent populations taking advantage of recent advances in treating the leading causes of premature death and recent gains in knowledge about health promotion. Arguing against such an interpretation, the decline in reducing premature mortality inequities started before current treatments reducing risk of premature mortality were available (e.g., antiretroviral drugs, statins, and surfactant for premature babies) [2,71,76] and before the marked decline of smoking among persons with greater education and affluence [72,73]. One potential additional contributing factor could accordingly be the adverse impact of post-1980 policies to “roll back” the welfare state [36–42,69,77]. These societal changes could conceivably have had an impact on premature mortality rates for many causes of death, as a consequence of policies that reduced federal responsibility and funds for public health and antipoverty programs (in part via “block grants”), froze the minimum wage, disproportionately decreased taxes on the wealthy (resulting in their growing concentration of wealth), and restricted affirmative action [36–42,69,77]. Also germane would be recent and related rising levels of medical uninsurance, persistent racial/ethnic and socioeconomic disparities in the quality of medical care, and delayed access of under-served populations to effective medical innovations [5,77,79]. We present these speculative interpretations as hypotheses to be tested, as opposed to verities that simply can be inferred from our data, and we suggest that they merit testing because of the high count and proportion of observed excess deaths and the implications for developing policies to avert health inequities.

In summary, our evidence of decreasing and then increasing or stagnating socioeconomic and racial/ethnic inequities in US premature mortality and infant death requires explanation and refutes the view that improvements in population health by default entail growing or shrinking health disparities, whether absolute or relative. Death is inevitable. Premature mortality is not.

## Supporting Information

Alternative Language Abstract S1 Translation of the Abstract into Spanish by Enrico Marcelli (26 KB DOC)

Figure S1 US Counties by Quintile of Median Household Income, 1960 (485 KB PDF)

Figure S2 US Counties by Quintile of Median Household Income, 1970 (488 KB PDF)

Figure S3 US Counties by Quintile of Median Household Income, 1980 (493 KB PDF)

Figure S4 US Counties by Quintile of Median Household Income, 1990 (501 KB PDF)

Figure S5 US Counties by Quintile of Median Household Income, 2000 (497 KB PDF)

Table S1 Cut-off Points for Maps of US County Median Family Income Quintiles: 1959, 1969, 1979, 1989, 1999 [51–53] (35 KB DOC)

## Abbreviations

CI: confidence interval
IRD: incidence rate difference
IRR: incidence rate ratio, NCHS, National Center for Health Statistics
PAF: population attributable fraction
