# Supplementary material for: The Fall and Rise of US Inequities in Premature Mortality: 1960–2002
Source: PLoS Med. 2008 Feb 26;5(2):e46. doi: 10.1371/journal.pmed.0050046 (PMC2253609; doi:10.1371/journal.pmed.0050046)
Supplement: Alternative Language Abstract S1 — (26 KB DOC) [file pmed.0050046.sd001.doc]

Resumen:

Antecedentes: Existe el debate sobre si una mejoría en la salud de la población en general trae consigo que la magnitud absoluta y relativa de las disparidades de salud se incrementen o se reduzcan. En correspondencia, decidimos probar la **hipótesis de que las desigualidades se amplian – o acortan – en un contexto de todos de mortalidad en descenso, examinando los datos de mortalidad annual en los Estados Unidos a lo largo de un período de 42 años.**

Metodología/principales hallazgos: Utilizando los datos de los censos decenales sobre la mortalidad por condados de Estados Unidos de 1960 a 2002 y la mediana del ingreso familiar por condado de 1960 al 2000, analizamos las tasas de mortalidad prematura (muerte antes de los 65 años) y mortalidad infantil (muertes de personas menores de un **año** de edad) por quintil de la mediana del ingreso familiar por condado ponderado por el tamaño de la población por condado.

Entre 1960 y 2002 encontramos que al reducirse en Estados Unidos la mortalidad prematura y las tasas de mortalidad infantil en todos los quintiles de los ingresos de los condados, las desigualdades socio económicas y étnicas/raciales en la mortalidad prematura y en la mortalidad infantil (tanto absoluta como relativa) disminuyeron entre 1966 y 1980, especialmente para la población de color en Estados Unidos. Las desigualdades relativas a la salud se agrandaron mientras que las diferencias absolutas apenas cambiaron en magnitud. Si entre 1960 y 2002 todas las personas hubieran experimentado las mismas tasas específicas de mortalidad por edad de la población blanca cuyo ingreso está situado en el quintil más alto, 14% de las muertes prematuras de los blancos y 30% de las muertes prematuras entre la gente de color no hubiera ocurrido.

Conclusiones/Importancia: las tendencias observadas refutan los señalamientos de que las desigualdades en salud inevitablemente se agrandan – o se reducen – cuando la salud de la población mejora. En lugar de ello, la magnitud de las **desigualidades de salud pueden bajar o elevarse, y es nuestro trabajo entender porgué.**
