# Supplementary material for: The Fall and Rise of US Inequities in Premature Mortality: 1960–2002
Source: PLoS Med. 2008 Feb 26;5(2):e46. doi: 10.1371/journal.pmed.0050046 (PMC2253609; doi:10.1371/journal.pmed.0050046)

US counties by quintile of median household income, 1960

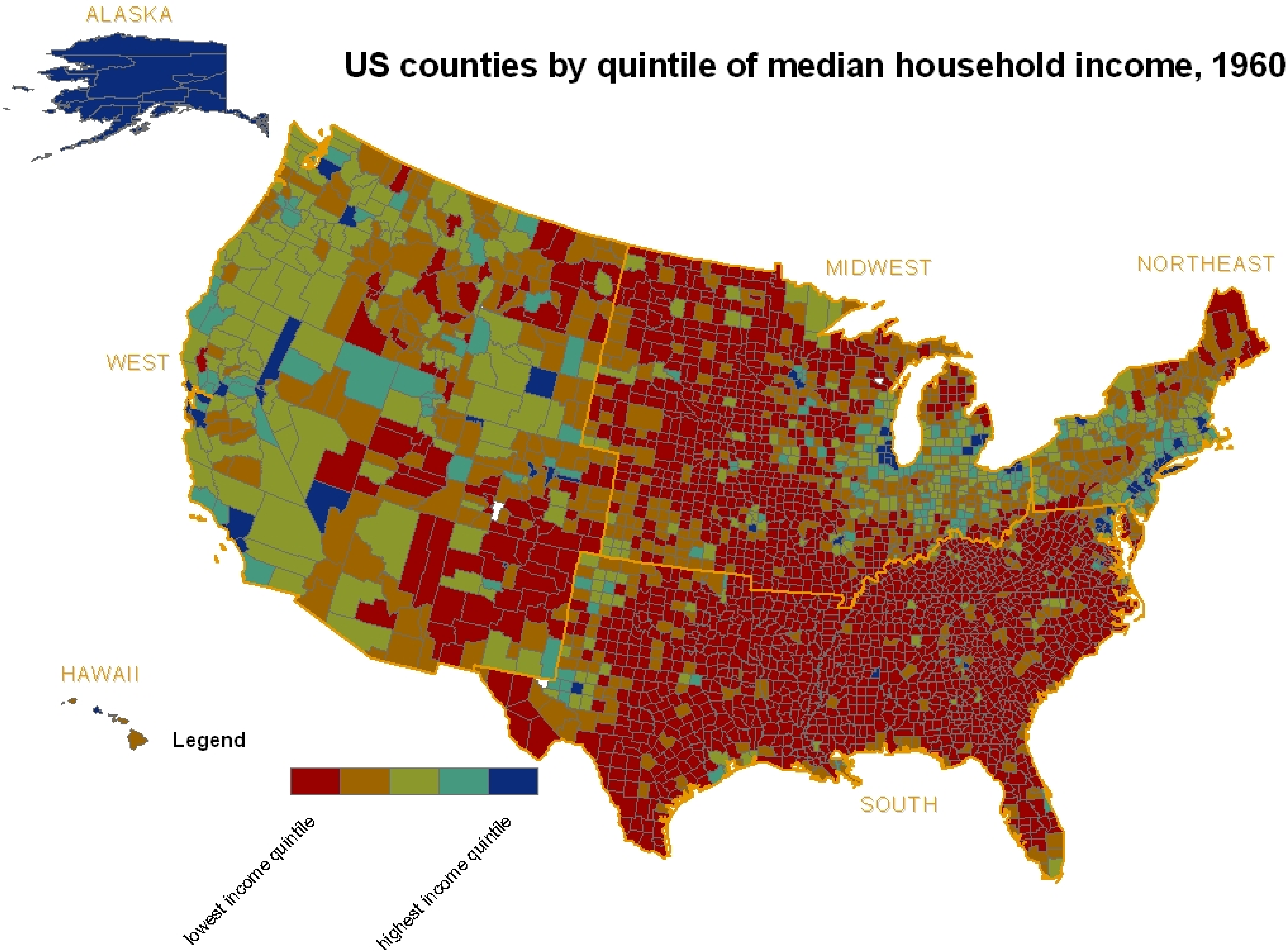

Supplement: Figure S1 — (485 KB PDF) [file pmed.0050046.sg001.pdf]
