# Supplementary figures and images for: The Fall and Rise of US Inequities in Premature Mortality: 1960–2002
Source: PLoS Med. 2008 Feb 26;5(2):e46. doi: 10.1371/journal.pmed.0050046 (PMC2253609; doi:10.1371/journal.pmed.0050046)

# US counties by quintile of median household income, 1990

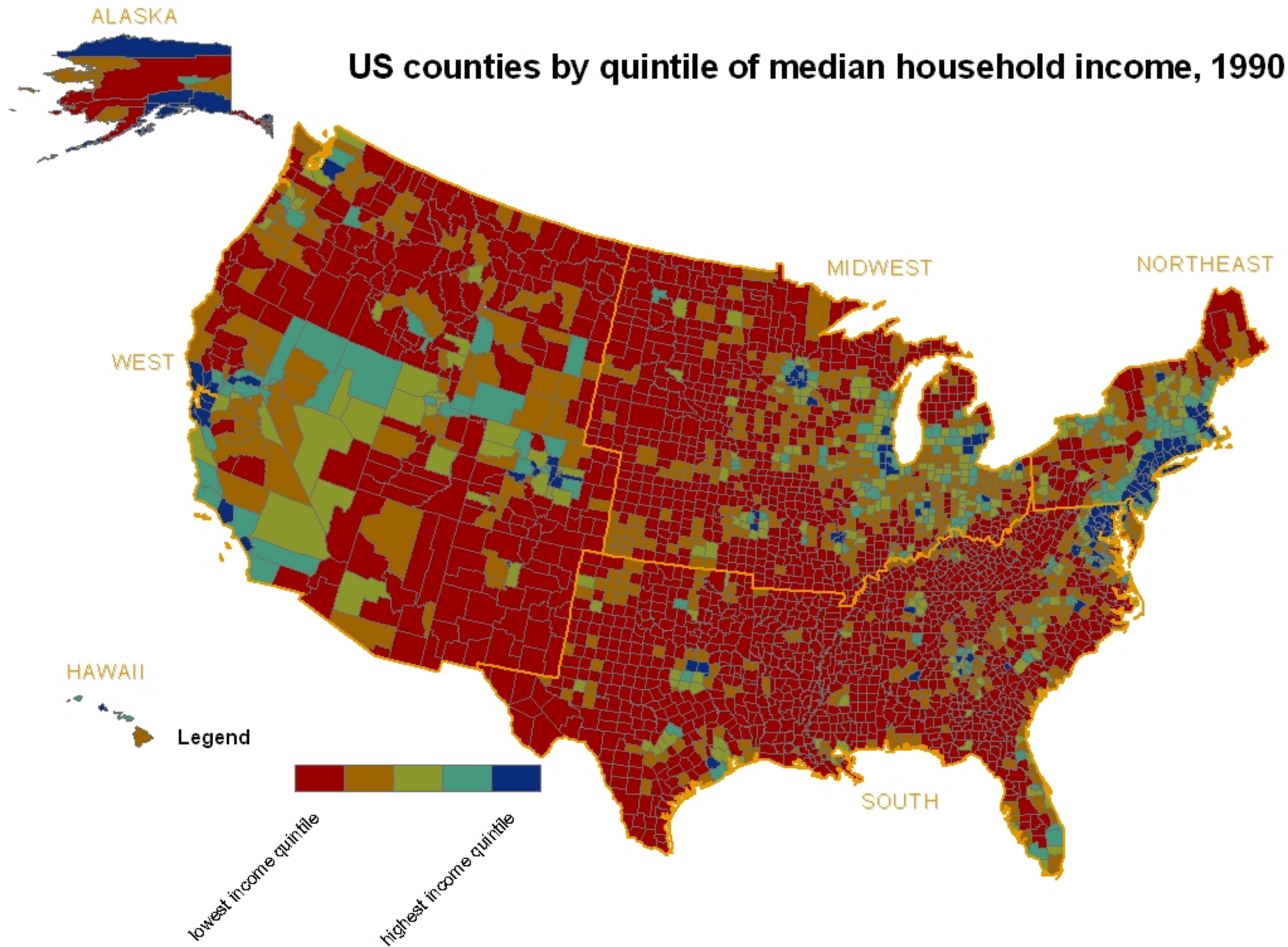

Supplement: Figure S4 — (501 KB PDF) [file pmed.0050046.sg004.pdf]

# US counties by quintile of median household income, 2000

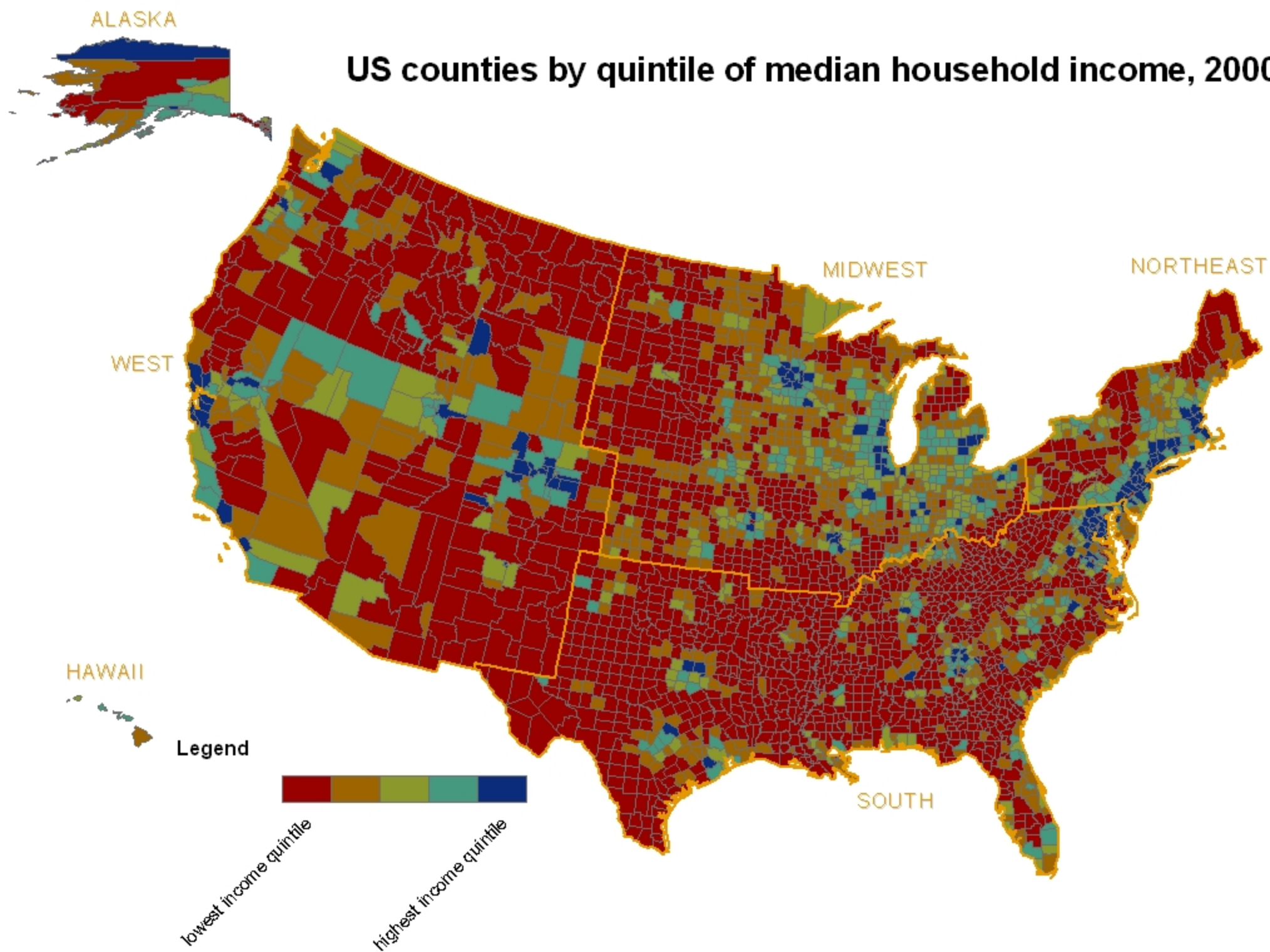

Supplement: Figure S5 — (497 KB PDF) [file pmed.0050046.sg005.pdf]
