# Supplementary material for: The Fall and Rise of US Inequities in Premature Mortality: 1960–2002
Source: PLoS Med. 2008 Feb 26;5(2):e46. doi: 10.1371/journal.pmed.0050046 (PMC2253609; doi:10.1371/journal.pmed.0050046)
Supplement: Table S1 — (35 KB DOC) [file pmed.0050046.st001.doc]

**Supporting Table S1.** **Cut-points and maps of US county median family income quintiles: 1959, 1969, 1979, 1989, 1999 [51-53]**

| **Year** | **County median family income quintile cut-points: upper bound for each quintile expressed in that year’s dollars** | | | | |
| --- | --- | --- | --- | --- | --- |
|  | **Quintile 1**  **(lowest)** | **Quintile 2** | **Quintile 3** | **Quintile 4** | **Quintile 5**  **(highest)** |
| 1960 | $12,020 | $15,437 | $18,300 | $21,552 | $38,445 |
| 1970 | $18,479 | $21,851 | $24,974 | $28,482 | $57,707 |
| 1980 | $24,522 | $28,021 | $31,232 | $35,342 | $73,665 |
| 1990 | $24,391 | $27,746 | $30,722 | $34,976 | $68,510 |
| 2000 | $36,125 | $40,301 | $44,449 | $50,813 | $101,823 |
